# Metabolic Patterns and Biotransformation Activities of Resveratrol in Human Glioblastoma Cells: Relevance with Therapeutic Efficacies

**DOI:** 10.1371/journal.pone.0027484

**Published:** 2011-11-11

**Authors:** Xiao-Hong Shu, Hong Li, Xiao-Xin Sun, Qian Wang, Zheng Sun, Mo-Li Wu, Xiao-Yan Chen, Chong Li, Qing-You Kong, Jia Liu

**Affiliations:** 1 Liaoning Laboratory of Cancer Genetics & Epigenetics, Department of Cell Biology, Dalian Medical University, Dalian, China; 2 Department of Medicinal Chemistry, College of Pharmacy, Dalian Medical University, Dalian, China; University of Nebraska Medical Center, United States of America

## Abstract

**Background:**

*Trans*-resveratrol rather than its biotransformed monosulfate metabolite exerts anti-medulloblastoma effects by suppressing STAT3 activation. Nevertheless, its effects on human glioblastoma cells are variable due to certain unknown reason(s).

**Methodology/Principal Findings:**

Citing resveratrol-sensitive UW228-3 medulloblastoma cell line and primarily cultured rat brain cells/PBCs as controls, the effect of resveratrol on LN-18 human glioblastoma cells and its relevance with metabolic pattern(s), brain-associated sulfotransferase/SULT expression and the statuses of STAT3 signaling and protein inhibitor of activated STAT3 (PIAS3) were elucidated by multiple experimental approaches. Meanwhile, the expression patterns of three SULTs (SULT1A1, 1C2 and 4A1) in human glioblastoma tumors were profiled immunohistochemically. The results revealed that 100 µM resveratrol-treated LN-18 generated the same metabolites as UW228-3 cells, while additional metabolite in molecular weight of 403.0992 in negative ion mode was found in PBCs. Neither growth arrest nor apoptosis was found in resveratrol-treated LN-18 and PBC cells. Upon resveratrol treatment, the levels of SULT1A1, 1C2 and 4A1 expression in LN-18 cells were more up-regulated than that expressed in UW228-3 cells and close to the levels in PBCs. Immunohistochemical staining showed that 42.0%, 27.1% and 19.6% of 149 glioblastoma cases produced similar SULT1A1, 1C2 and 4A1 levels as that of tumor-surrounding tissues. Unlike the situation in UW228-3 cells, STAT3 signaling remained activated and its protein inhibitor PIAS3 was restricted in the cytosol of resveratrol-treated LN-18 cells. No nuclear translocation of STAT3 and PIAS3 was observed in resveratrol-treated PBCs. Treatment with STAT3 chemical inhibitor, AG490, committed majority of LN-18 and UW228-3 cells but not PBCs to apoptosis within 48 hours.

**Conclusions/Significance:**

LN-18 glioblastoma cells are insensitive to resveratrol due to the more inducible brain-associated SULT expression, insufficiency of resveratrol to suppress activated STAT3 signaling and the lack of PIAS3 nuclear translocation. The findings from PBCs suggest that an effective anticancer dose of resveratrol exerts little side effect on normal brain cells.

## Introduction

Glioblastoma multiforme (GM) is the most common primary brain malignancy in human adults [1]. Irrespective to the combination of surgical operation with improved external radiotherapy and adjuvant chemotherapy, the prognosis of GMB remains very poor due to its highly aggressive biological behavior and frequent recurrence rate [2]. Therefore, exploring effective and less toxic chemotherapeutic approaches would be of clinical values in better management of this sort of lethal disease. So far, the data about the effects of resveratrol on some human GM cell lines are promising [3]–[5]. However, it remains largely unknown whether resveratrol encounters resistance in GM cells and how normal brain cells respond to this agent.

Resveratrol is a polyphenolic phytoalexin possessing a variety of biological activities [6]–[8]. It is still obscure whether *trans*-resveratrol or its metabolite(s) exerting the therapeutic effects [9], however, this issue has been elucidated at least in part by our recent findings from human medulloblastoma (MB) cells [10], which revealed that *trans*-resveratrol was mainly biotransformed to resveratrol monosulfates in MB cells but *trans*-resveratrol rather than its monosulfate exerted anti-MB effects. The above findings indicated that the declined sulfonation activity might prolong the intracellular bioavailability and sustain anticancer efficacy of *trans*-resveratrol. To gain new insight into this issue, it would be necessary to further address whether the metabolic pattern and/or the metabolic activity of resveratrol are different in the sensitive and resistant resveratrol treated cancer cells.

Citing UW228-3 MB cells as resveratrol-sensitive control and primarily cultured 1-day-old Wistar rat brain cells (PBCs) as normal control [11], the effects of resveratrol and its relevance with metabolic pattern(s) and metabolite-associated gene expression in LN-18 GM cells were evaluated after the cells were treated with resveratrol for 48 hours. Since STAT3 signaling was considered as the main molecular target of resveratrol in MB cells [12] and the important survival factor for GM cells [13], [14], the status of this signaling, the expression of protein inhibitor of activated STAT3 (PIAS3) and the effects of STAT3 chemical inhibitor AG490 on LN-18 cells was investigated and the results were compared with that obtained from UW228-3 and PBC cells.

## Results

### Differential resveratrol sensitivities

**Figure 1:**
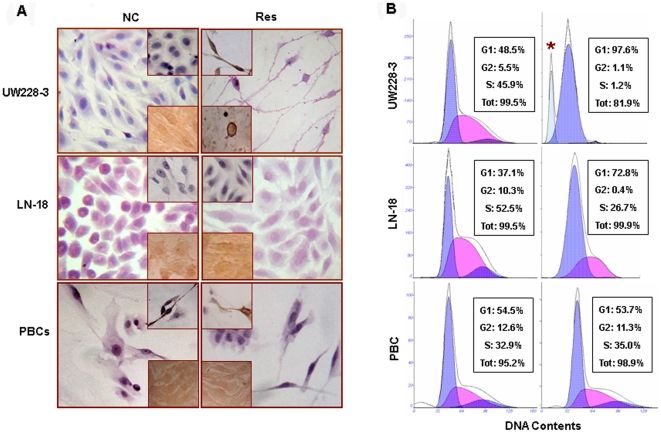
Determination of resveratrol sensitivities of LN-18 and PBC cells to resveratrol. (A) H&E morphological staining performed on UW228-3, LN-18 and PBC cells without (normal culture) and with 100 µM resveratrol treatment for 48 hours. The results of synaptophysin-oriented ICC and TUNEL assay are shown in the upper and the lower insets for each of the main images, respectively. (B) Flow cytometry fractionation of cell cycles and apoptotic cells in UW228-3, LN-18 and PBC cell populations without (NC) and with (Res) 100 µM resveratrol treatment for 48 hours. *, indicates the peak of apoptotic cells.

As shown in Figure 1A, LN-18 cells were spindle-like in shape, which showed neither morphologic change nor apoptotic cell death after 100 µM resveratrol treatment for 48 hours. Similarly, 100 µM resveratrol treated primarily cultured rat brain cells including neurons and glial cells kept growth and showed no sign of cell death. As the resveratrol-sensitive control, elliptical UW228-3 cells showed neuron-like morphology, synaptophisin expression (upper insets for Figure 1A) and distinct apoptosis hallmarks (lower insets for Figure 1A). 0.25% trypan blue viable/nonviable cell discrimination assay revealed that after 100 µM resveratrol treatment for 0, 12, 24, 36, and 48 hours, the percentage of nonviable cells was 0.16%, 11.23%, 30.9%, 35.46%, and 42.86% in UW228-3 cells, 0.19%, 1.21%, 5.63%, 8.71%, and 10.50% in LN-18 cells and 0.21%, 1.19%, 1.66%, 1.83%, and 2.67% in PBC cells. FCM analyses (Figure 1B) further demonstrated that G1 and S fractions were 48.5% and 45.9% in normal UW228-3 cells and changed to 97.6% and 1.2% in the resveratrol-treated cells. The percentages of G1 and S phase LN-18 cells were 37.1% and 52.5% under normal culture condition and became 72.8% and 26.7% after 100 µM resveratrol treatment for 48 hour. In PBC cells, G1 and S fractions were 54.5% and 32.9% in normal cells and remained almost unchanged (53.7% and 35.0%) after resveratrol-treatment. The percentages of apoptotic cells in 100 µM resveratrol-treated UW228-3, LN-18 and PBC cells were 18.1%, 0.1% and 1.1%, respectively. Because of the G1 arrest of resveratrol-treated LN-18 cells and almost unchanged cell cycle fractions in resveratrol-treated PBCs, LN-18 can be regarded as resveratrol-insensitve and PBCs as resveratrol-resistant cells.

### Resveratrol monosulfate detection in LN-18 cells

**Figure 2:**
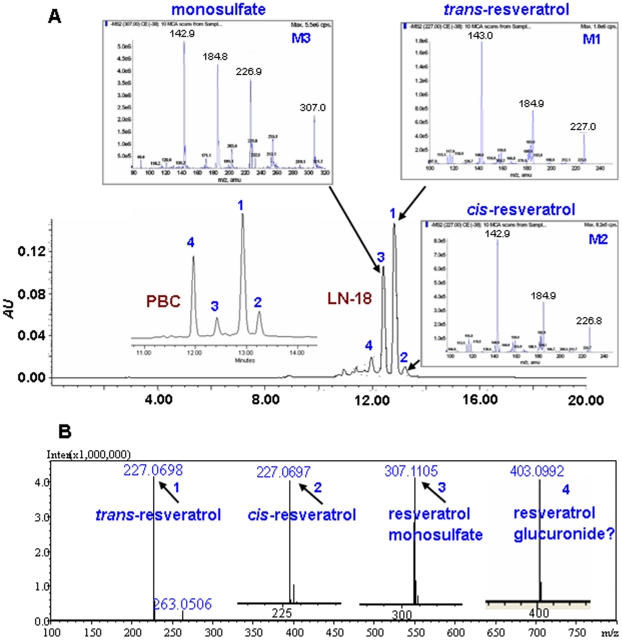
The metabolic patterns analyses of resveratrol in LN-18 and PBC cells. (A) The MS/MS spectrum of M1, M2 and M3 correspond to 1, 2 and 3 of the HPLC peaks. 4, an unidentified compound produced predominantly in PBCs. (B) Shimadzu LCMS-IT-TOF-based HRMS analysis of resveratrol metabolites in LN-18 cells and PBCs. Arrows labeled as *trans*-resveratrol, *cis*-resveratrol, resveratrol monosulfate and resveratrol glucuronide? indicate the exact [M–H]^-^ molecular ion weight of 227.0698 (C_14_H_11_O_3_, calculated m/z 227.0708), 227.0697 (C_14_H_11_O_3_, calculated m/z 227.0708), 307.1105 (C_14_H_11_SO_6_, calculated m/z 307.0276) and 403.0992 (C_20_H_19_O_9_, calculated m/z 403.1035), respectively.

To identify the resveratrol metabolites in LN-18 cells, the cells and the condition media were collected separately after 48-hour resveratrol treatment, cleaned up by SPE to eliminate the interferer and subjected to HPLC and LC-MS/MS analyses. Resveratrol-containing cell-free medium and the same kinds of samples from UW228-3 and PBC cells were used as controls. HPLC analyses revealed three major compounds in LN-18 cell lysate and condition medium, which were resveratrol standard (*trans*-resveratrol), *cis*-resveratrol and resveratrol monosulfate as that found in UW228-3 samples [10], according to their retention time in HPLC (Figure 2A), molecular weight in MS and HRMS (Figure 2B) [15]. In addition to the above three compounds, another major metabolite in the retention time of 11.96 minutes (*t_R_* = 11.96) and molecular weight of 403.0992 in negative ion mode was detected in the lysate and condition medium of PBC cells (Figure 2A), suggesting the variable resveratrol metabolic patterns between rat and humans.

### Resveratrol upregulated SULT expression

**Figure 3:**
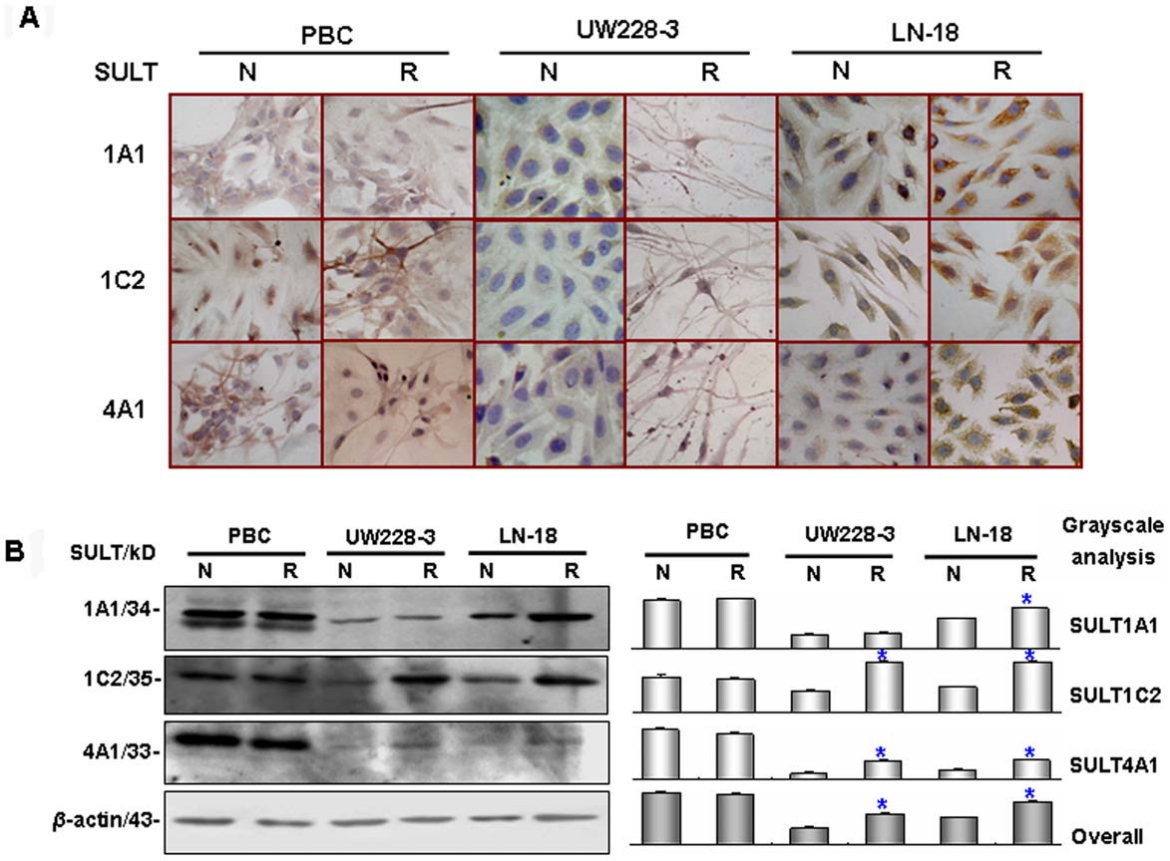
Resveratrol upregulated SULT1A1, 1C2 and 4A1 expression. (A) ICC evaluation of SULT1A1, 1C2 and 4A1 expression in PBC, UW228-3 and LN-18 cells without (N) and with (R) resveratrol treatment. (B) Western blot analyses of SULT1A1, 1C2 and 4A1 expression in LN-18 cells without (N) and with (R) resveratrol treatment and compared with that in normal control PBCs and resveratrol-sensitive control medulloblastoma UW228-3 cells. *β*-actin was used as loading control and for calculation of SULT expression levels/densitometry scan of Western blotting results. *Compared with normal cultured LN-18 and UW228-3 cells, respectively; *represents statistical significance (*p*<0.05).

Since SULT1A1, 1C2 and 4A1 were preferably expressed in human and rodent brains [16]–[18], the responsibility of the three SULTs for resveratrol sulfonation was evaluated by checking their statuses in LN-18 cells before and after resveratrol treatment by immunocytochemical (ICC) staining and Western blotting. As shown in Figure 3, the three brain-associated SULT1A1, 1C2 and 4A1 were expressed in high levels in the PBC cells, and the reverse also held true in UW228-3 cells. In the case of LN-18 cells, SULT1A1 and 1C2 were expressed in low and SULT4A1 in extremely low levels. After resveratrol treatment, SULT4A1 became detectable, SULT1C2 and, especially, SULT1A1 were enhanced in LN-18 cells (Figure 3). The densitometry scan of Western blot results revealed that the total three SULT level in resveratrol-treated LN-18 cells was about 1.63 folds higher than that produced by its normally cultured counterpart, 1.44 folds higher than that in resveratrol-treated UW228-3 cells and about 86.3% as much as that in resveratrol-treated PBC cells (Figure 3B). Although SULT1A1, 1C2 and 4A1 levels were enhanced as well in resveratrol-treated UW228-3 cells, the extents were 31.6% lower than that of LN-18 cells (Figure 3B).

### Variable brain-associated SULT expression in GM tissues

**Figure 4:**
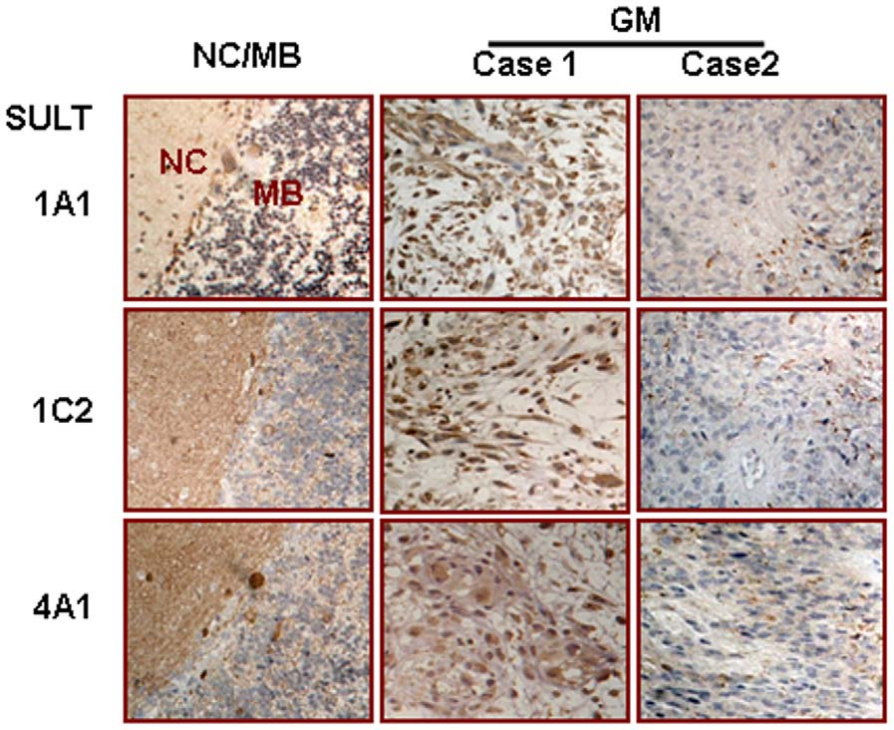
The expression of SULT1A1, 1C2 and 4A1 in glioblastomas tissues. Immunohistochemical illustration of differential expression patterns of SULT1A1, 1C2 and 4A1 in two cases of glioblastomas (GM). Medulloblastoma (MB) and its surrounding noncancerous cerebellum tissue (NC) were cited as controls.

TMA-based immunohistochemical (IHC) staining showed that SULT1A1, 1C2 and 4A1 expression in the brain tissues surrounding MBs and GMs were as high as the levels in normal rat brain, while in the case of GMs, the levels of SULT1A1, 1C2 and 4A1 expression were highly variable (Figure 4A; Table 1). The expression patterns of these SULT genes among 149 GM cases and 48 tumor-surrounding brain tissues were classified as undetectable (−), decreased (+) and unchanged levels (>++) and their frequencies were summarized in Table 1. It was found that of 149 GM cases, 42.0%, 27.1% and 19.6% showed similar SULT1A1, 1C2 and 4A1 levels (>++) as that of tumor-surrounding tissues and the remaining parts showed reduction (+) and even absence (−) of SULT1A1, 1C2 and 4A1 expression.

**Table 1:** Immunohistochemical profiling of SULT expression in human brain tissues.

Samples	No.	SULT1A1 (%)			SULT1C2 (%)			SULT4A1 (%)		
		- $	+	>++	-	+	>++	-	+	>++
**Tissue specimens**	197									
Noncancerous	48	0 (0)	10 (62.5)	6 (37.5)	0 (0)	7(43.7)	9(56.3)	0 (0)	0 (0)	16 (100)
glioblastomas	149	5 (10.0)	24(48.0)	21 (42.0)	4 (8.3)	31 (64.6)	13(27.1)*	27(52.9)	14(27.5)	10 (19.6)*

*represents statistical significance (*p*<0.05).

*compared with noncancerous tumor surrounding tissues.

$-, undetectable immuno-labeling; +, positive but decreased immuno-labeling; ++, strong immuno-labeling.

### STAT3 and PIAS3 in the three types of cells

**Figure 5:**
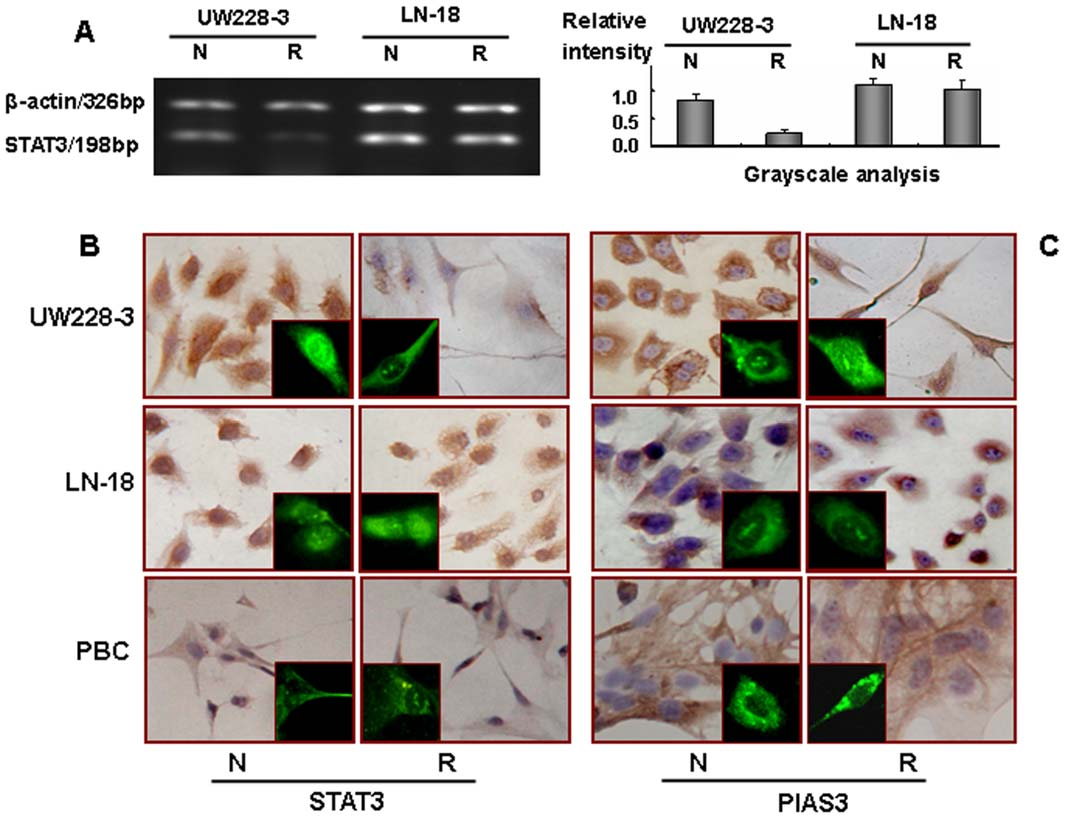
Evaluation of STAT3 and PIAS3 statuses in UW22-3, LN-18 and PBC cells. STAT3-oriented RT-PCR (A), immunocytochemical and immunofluorescent illustration of intracellular distribution of STAT3 (B) and its inhibitor PIAS3 (C) in LN-18 cells without (N) and with (R) 100 µM resveratrol treatment. Immunofluorescence results were shown in the insets for Fig 5B and 5C. Corresponding data obtained from PBC and UW228-3 cells were used as controls.

The potential influence of resveratrol in STAT3 signaling was evaluated by checking the expression and intracellular distribution of STAT3 and PIAS3. As shown in Figure 5, STAT3 signaling was in activated status in normally cultured LN-18 cells because of the presence of STAT3 in both cytosolic space and the nuclei, and this situation remained unchanged after 48 hours resveratrol treatment (Figure 5B). Similarly, PIAS3 was constitutively expressed and distributed in the cytoplasm regardless of resveratrol treatment (Figure 5C). In resveratrol-sensitive UW228-3 cells, STAT3 expression was down-regulated accompanied with infrequent STAT3 nuclear translocation (insets for Figure 5A and 5B); reversely, PIAS3 was found in the cytoplasm of UW228-3 cells but translocalized into the nuclei upon resveratrol treatment (insets for Figure 5C). In the case of PBCs, both STAT3 and PIAS3 were localized in cytosol regardless of resveratrol treatment (Figure 5B and 5C).

### AG490 caused growth arrest and apoptosis of LN-18 but not PBCs cells

**Figure 6:**
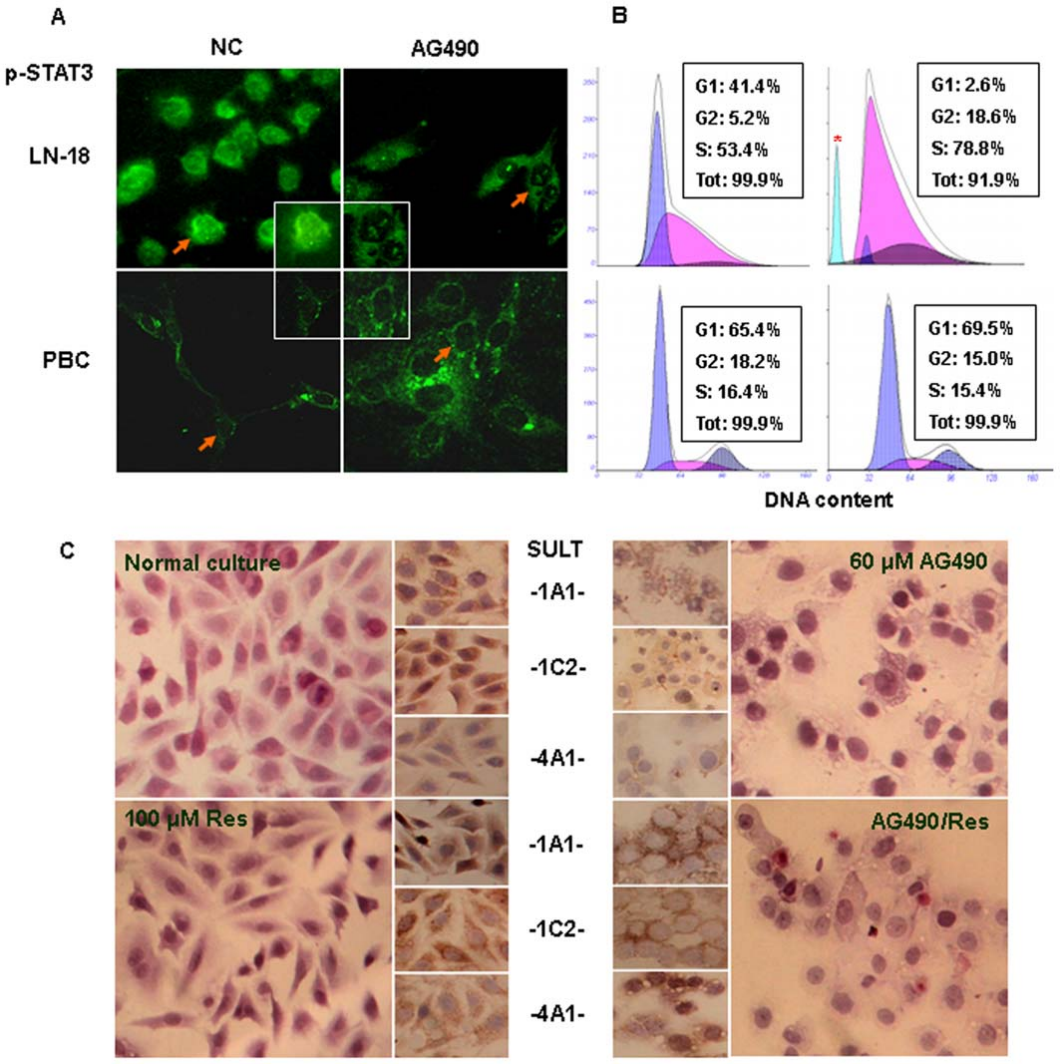
Sensitivities of LN-18 and PBC cells to STAT3 inhibitor AG490. (A) Immunofluorescence illustration of intracellular distribution of STAT3 in LN-18 and PBC cells without (N) and with (AG490) 60 µM AG490 treatment. Arrows indicate the cells shown in the insets in high magnification (X400). (B) Flow cytometry analysis revealed reduction of G1-phase cells, accumulation of S-phase cells and induction of apoptosis (blue peak) in AG490-treated LN-18 cell population. The cell cycle progression was almost unchanged in PBC cells. *, indicates the peak of apoptotic cells. (C) H&E morphologic examination of LN-18 cells under normal culture or incubated with 100 µM *trans*-resveratrol, 60 µM AG490 or resveratrol/AG490 mixture for 48 hours (Main images). Small images: immunocytochemical illustration of SULT1A1, 1C2 and 4A1 expression in LN-18 cells treated by 60 µM AG490 without/with 100 µM resveratrol supplementation. Normally cultured and 100 µM resveratrol-treated LN-18 cells were cited as controls.

To determine the correlation of the unchanged STAT3 activation with resveratrol insensitivity of human GM cells, 60 µM AG490, a selective inhibitor of STAT3 phosphorylation, was used to treat LN-18, UW228-3 and PBC cells, respectively. ICC staining of LN-18 cells demonstrated that as similar as the situation in UW228-3 cells (data not shown), STAT3 nuclear translocation was distinctly inhibited by AG490 (Figure 6A), accompanied with remarkably decreased fraction of G1 phase cells (2.6%) and increased apoptotic cell death (8.1%; Figure 6B). In contrast, AG490 exerted little effect on the growth and survival of PBCs (Figure 6). Additionally, LN-18 cells treated with AG490 and resveratrol combination (AG490/Res) showed more or less up-regulated SULT1A1, 1C2 and 4A1 expression but did not undergo more severe cell crisis than that caused by AG490 alone (Figure 6C **)**, suggesting the importance of STAT3 signaling in the survival of LN-18 cells, STAT3 signaling as the major molecular target of resveratrol and no influence of STAT3 inhibition in reseveratrol regulated SULT expression.

## Discussion

*Trans-*resveratrol has been increasingly recognized as an ideal cancer therapeutic agent, because of its nontoxic property and cancer preventive activities in a variety of human and rodent cancers [19], [20]. *Trans*-resveratrol can be biotransformed intracellularly, generating one or more metabolites in cell type related patterns [21]–[23]. Therefore, determination of the bioactive form(s) of resveratrol in individual cell and cancer types becomes a fundamental issue for successful application of this compound in preventive and clinical medicine. In this context, a comparison of resveratrol metabolic patterns in resveratrol-sensitive and -resistant cancer cells would be helpful to figure out this issue. MB cells including UW228-3 were sensitive to resveratrol [10], [11]. Our current study clearly demonstrates for the first time that human LN-18 GM cells are insensitive and rat PBC cells are resistant to resveratrol. The distinct responses of these three types of cells to resveratrol thus provides an ideal model to shed light on the underlying reasons leading to different therapeutic outcomes of resveratrol in malignant and normal brain cells.

It was found that *trans*-resveratrol rather than its biotransformed form, resveratrol monosulfate, exhibited anti-MB effects and that the metabolic activity mediated by brain-associated SULTs was declined in MB cells *in vivo* and *in vitro* [10]. We therefore supposed that the metabolic pattern and/or metabolic efficiency of resveratrol might be different between resveratrol-sensitive and -resistant cancer cells. To clarify this point, the combination of HPLC, LC-MS/MS and HRMS techniques was used to compare the resveratrol metabolite(s) in UW228-3, LN-18 and PBC cells. Like the situation in resveratrol-sensitive UW228-3 cells, resveratrol monosulfate is identified as the major metabolite in LN-18 cells. Although both *trans-* and *cis-*resveratrol can be metabolized to monosulfate forms [22], [24], the monosulfate metabolite in LN-18 cells may be mainly generated from trans-resveratrol, because we prepared trans-resveratrol solution freshly and handled it carefully during the experiment to minimize *trans-* to *cis*-resveratrol transition. Resveratrol-resistant PBC cells shared similar metabolites with the above two cell lines, but a compound in the retention time of 11.96 minutes rather than resveratrol monosulfate became predominant in the cell lysate and condition medium. Although the additional metabolite of molecular weight of 403.0992 in negative ion mode remains to be further characterized, the above findings suggest that the resveratrol metabolic pattern by itself seems to follow a tissue- and even species-specific fashions, therefore, may be unnecessarily related with the chemosensitivities of primary brain tumors.

Resveratrol monosulfate is the metabolite of phase II sulfonation reaction mediated by SULTs. The anti-MB effects of 100 µM *trans*-resveratrol were apparently attenuated when a great fraction of it was biotransformed to monosulfate [10], [22], reflecting a reverse relationship of SULT expression with resveratrol bioactivity. This notion has been further supported by our more recent finding that rat PBCs even tolerate as high as 200 µM resveratrol treatment (Shu XH et al. Unpublished data). Since SULT1A1, 1C2 and 4A1 are preferably expressed in the rodent brain [16]-[18], their expression levels in LN-18 without and with resveratrol treatment was analyzed and compared with that in UW228-3 cells and PBC cells. Although the baseline levels of the three brain-associated SULTs in LN-18 and UW228-3 cells were much lower than that in PBC cells, SULT1A1 and 1C2 upregulation was more distinct in resveratrol-treated LN-18 cells. This phenomenon indicates 1) that the brain-associated SULTs may be more upregulatable in GM cells and 2) that the sulfonation activity in LN-18 cells would be higher than that in UW228-3 cells. In agreement with the *in vitro* findings, tissue microarray-based IHC for the three brain-associated SULTs revealed diverse SULT expression patterns in 149 GM cases of which 42.0%, 27.1% and 19.6% showed similar levels of SULT1A1, 1C2 and 4A1 as that of tumor surrounding tissues. Since SULT-mediated sulfonation reduces resveratrol bioactivity via modifying resveratrol's chemical structure, the unchanged *in vivo* expression levels and more upregulatable features of SULTs may be unfavorable for maintaining an effective dose of resveratrol in GM cells. In agreement with this notion, the overall SULT level of resveratrol-resistant PBC cells was high and two types of resveratrol metabolites were generated.

Resveratrol possesses multiple molecular effects on cancer cells in dose-dependent fashion and STAT3 signaling is one of its molecular targets [12], [25]. The case also holds true in resveratrol-treated MB cells in which STAT3 signaling and its downstream gene expression are concurrently inhibited [26]. Since STAT3 signaling is critical for both MBs [12] and GMs [27], the statuses of this signaling in LN-18 and PBC cells without and with resveratrol treatment were elucidated. It was revealed that STAT3 activation was found in LN-18 but not PBC cells under normal culture condition. Unlike the situation in UW228-3 cells, STAT3 signaling in resveratrol-treated LN-18 cells remained activated in terms of the presence of nuclear translocation and constant expression of STAT3. These data indicates that the lack of response of this signaling to resveratrol in LN-18 cells may be linked with resveratrol insensitivity and the insufficient intracellular *trans*-resveratrol bioavailability. This speculation is further supported by the following findings: 1) neither STAT3 activation nor cellular crisis is observed in PBC cells with high resveratrol biotransformation activities and 2) AG490 by itself is sufficient to induce significant cell death of LN18 cells.

Protein inhibitor of activated STAT3 (PIAS3) negatively regulates STAT3 activation [28], [29] and the loss of PIAS3 in GM cells contributes to enhanced STAT3 transcriptional activity and subsequent cell proliferation [28], [30]. Therefore, the relevance of PIAS3 with resveratrol cytotoxic effects was investigated by the use of UW228-3, LN-18 and PBS cells. The results revealed that PIAS3 was expressed in those cells and distributed in the cytoplasm under normal culture condition. On exposure to resveratrol, apparent PIAS3 nuclear translocation accompanied with reduced STAT3 nuclear immuno-labeling was observed in resveratrol-sensitive UW228-3 cells, while PIAS3 was restricted in the cytosolic space of resveratrol-insensitive LN-18 cells regardless of STAT3 nuclear translocation. In the case of resveratrol-resistant PBCs, neither PIAS3 nor STAT3 showed nuclear translocation. Since PIAS3 exerts a profound inhibitory effect on STAT3-mediated transcription of target promoters in nucleus [29], our findings indicate a potential correlation of PIAS3 nuclear translocation or the presence of nuclear STAT3 with the fate of resveratrol-treated cells. Therefore, it is reasonable to suppress proliferation and induce apoptosis of LN-18 cells but not PBCs with STAT3 chemical inhibitor, AG490.

Taken together, our current study demonstrates that LN-18 GM cells share similar resveratrol metabolites with UW228-3 MB cells regardless of the differences of their resveratrol sensitivities. The expression levels of brain-associated SULTs are higher in resveratrol-insensitive LN-18 and especially PBC cells than that in resveratrol-sensitive UW228-3 MB cells. Consequently, the relatively high efficiency of *trans*-resveratrol biotransformation, the lack of response of STAT3 signaling to resveratrol and the rarity of nuclear translocation of PIAS3 may confer resveratrol-insensitive properties on LN-18 cells. Given the evidence of differential SULT expression patterns in human GMs, evaluations of metabolic activity and the sensitivity of STAT3 signaling to resveratrol would be of potential values in individualized application of resveratrol in the management of GMs. The findings from PBCs suggest that an effective anticancer dose of resveratrol has little side-effect on normal brain cells.

## Materials and Methods

### Primary rat brain cell culture

Two 1-day-old Wistar rats were obtained from the Experimental Animals Center of Dalian Medical University. The rat brains were freshly removed and minced with a scalpel and triturated in high glucose DMEM (Gibico, Invitrogen Corporation, Grand Island, NY, USA). After centrifugation at 2000 rpm for 5 minutes, the brain cells were washed with DMEM and centrifugated for 5 minutes. The cell suspensions were plated to 60 mm dishes (Nunc A/S, Rosklide, Denmark) and cultured in high glucose DMEM supplemented with 10% FBS under 37°C and 5% CO_2_ condition. 14 days later, the cells were used for experimental purposes. All experimental protocols had been reviewed and approved by the ethics committee of Dalian Medical University (ECDMU-09066) for protection of human subjects and experimental animals before conducting the project.

### Cellular effects of resveratrol

LN-18 human GM cell line [31] and UW228-3 human MB cell line [32] were cultured in DMEM (Gibco, USA) containing 10% fetal bovine serum (Gibco, USA) under 37°C and 5% CO_2_ condition. UW228-3, LN-18 cells and PBCs were treated by 100 µM resveratrol respectively for 48 hours. The total cell numbers and viability of the three cell lines with or without resveratrol treatment were determined with 0.25% trypan blue (Sigma Chem Co., St. Louis, MO, USA). Meanwhile, cell-bearing coverslips were harvested and fixed properly for H&E staining (hematoxylin and eosin staining), TUNEL (terminal deoxynucleotidyl transferase mediated nick end labeling; Promega Corp., Madison, WI, USA) assay and immunocytochemical staining for SULTs and neuronal differentiation marker synaptophisin. FCM (flow cytometry) was conducted to evaluate the effects of resveratrol on cell growth and survival. The cells cultured in conventional medium with 0.2% DMSO supplementation were used as controls. Each of experimental groups was set in triplicate, and the experiments were repeated at least three times to establish confidential conclusions.

### Identification of resveratrol metabolites

After 100 µM resveratrol treatment for 48 hours, the cell lysates and the conditional media were prepared from LN-18, UW228-3 and PBC cells by the methods described elsewhere [10]. They were purified and concentrated by Cleanert PEP-SPE cartridges (60mg; Agela Technol Inc. PA, USA) for HPLC analysis. Structural identification of resveratrol metabolite(s) generated by resveratrol-treated cells was performed on cell lysates and condition media respectively using LC/MS system (Applied Biosystem/MDS SCIEX, Foster City, CA, USA). Accurate masses were conducted by the use of high resolution mass spectrometry (HRMS, Shimadzu Co., Kyoto, Japan) analysis [10].

### *In vitro* and *in vivo* expression of brain-associated sulfotransferases (SULTs)

ICC staining and Western blot analysis were performed on the samples obtained from each of the experimental groups using the rabbit anti-human SULT1A1, 1C2 and 4A1 antibodies (ProteinTech Group, Inc., Chicago, USA) by the method described elsewhere [10]–[12]. The results were compared with that obtained from UW228-3 MB cells (as resveratrol-sensitive control) and PBCs (as normal control).

For IHC staining, 149 GM surgical specimens were collected from the First Affiliated Hospital of Dalian Medical University and Anshan Central Hospital, Anshan, China, after getting patients' consent. They were incised from the tumor mass and, where possible, tumor-surrounding tissues. The SULT1A1, 1C2 and 4A1 were selected for IHC because of their preferential expression in brain [16]–[18]. At least three pathologists blind to the sample are involved in the semi-quantitative analysis of the staining results according to the labeling intensity, and scored as negative (−) if no immuno-labeling was observed in target cells, weakly positive (+) if the labeling was faint, and moderately to strongly positive (>++) when the labeling was stronger or distinctly stronger than (+).

### Evaluation of STAT3 activation and PIAS3 expression

Activated STAT3 signaling is crucial for the growth and survival of both MBs and GMs [33], [34] and is supposed to be the major molecular target of resveratrol [35]. Therefore, the potential influence of resveratrol in STAT3 signaling was evaluated by checking the expression and intracellular distribution of STAT3 and its protein inhibitor PIAS3 in LN-18 cells before and after resveratrol treatment. ICC and immunofluorescence (IF) staining were performed on the coverslips obtained from each of the experimental groups. The antibodies against STAT3 and PIAS3 were used according to the manufacturer's instruction (Santa Cruz Biotech, Inc, CA, USA). Meanwhile, total cellular RNAs were prepared from LN-18 cells without and with resveratrol treatment for paralleled RT-PCR by the use of STAT3 primers (f: 5′-GGGTGGAGAAGGACATCAGCGGTAA-3′ and r: 5′-GCCGACAATACTTTCCGAATGC-3′) and µ-actin (f: 5′-GCATGGAGTCCTGTGGCAT-3′ and r: 5′-CATGAAGCATTTGCGGTGG-3′) [12]. The corresponding samples obtained from UW228-3 cells were used as control.

### Inhibition of STAT3 activation with AG490

AG490 (Sigma), a JAK_2_-specific inhibitor, was dissolved in DMSO to a stock concentration of 50 mM and was diluted to the final concentration of 60 µM with conventional culture medium just before use. Four experimental groups were set as follows: Group 1, normal culture; Group 2, 2‰ DMSO treatment as background control; Group 3, treatment with 60 µM AG490; Group 4, combination treatment with 60 µM AG490 and 100 µM resveratrol. For morphologic evaluation, ICC and IF staining, the coverslips were put into the dishes before initial cell seeding and were collected after 48 hours treatment, using the antibodies against STAT3 (Santa Cruz, CA). FCM was conducted to evaluate the effects of AG490 on cell growth and survival.

### Statistical analyses

The experimental data were expressed as mean ± SD. Mann-Whitney tests were used to analyze the statuses of SULT1A1, 1C2 and 4A1 expression in different histological groups and expressed in *p*-values. Statistical significance can be established if the *p*-value is less than 0.05.
